# Prognostic value and clinicopathological characteristics of PD-L1 overexpression in non-Hodgkin lymphoma: a meta-analysis

**DOI:** 10.1186/s12885-020-6550-z

**Published:** 2020-01-28

**Authors:** Qiang Zeng, Zhigang Liu, Ting Liu

**Affiliations:** Department of Hematology, West China Hospital, Sichuan University, Chengdu, 610041 China

**Keywords:** PD-L1, Non-Hodgkin lymphoma, Prognosis, Meta-analysis

## Abstract

**Background:**

Programmed cell death ligand 1 (PD-L1) has already been detected in various carcinomas. In non-Hodgkin lymphoma (NHL), however, the prognostic value of PD-L1 overexpression remains unclear.

**Methods:**

A meta-analysis of 2321 NHL patients from 12 studies was performed. Hazard ratios (HRs) with 95% confidence intervals (CIs) were used to evaluate the correlation between PD-L1 overexpression and prognosis of NHL, and odds ratios (ORs) with 95% CIs were used to assess the association of PD-L1 overexpression with clinicopathological factors.

**Results:**

The results showed that no significant difference between PD-L1 positive and negative groups was detected in NHL (HR: 1.40, 95% CI: 0.90–2.19; *P* = 0.137). Nevertheless, the results indicated that PD-L1 overexpression was associated with poor prognosis in the subtype of diffuse large B cell lymphoma (DLBCL) (HR: 1.70, 95% CI: 1.05–2.74; *P* = 0.031). We also performed subgroup analyses and meta-regression. The pooled OR showed that PD-L1 overexpression was associated with B symptoms, higher international prognostic index (IPI) score (3, 4, and 5 points) and Ann Arbor Stages III and IV.

**Conclusions:**

The meta-analysis demonstrated that PD-L1 expression was not associated with prognosis of NHL but was associated with prognosis of DLBCL.

## Background

Non-Hodgkin lymphoma (NHL), accounting for approximately 90% of lymphomas and comprising various subtypes, is a common hematological tumor. NHL is characterized by a series of malignant DNA repair obstacle events and activating proto-oncogene caused by viral or bacterial infection, immune dysfunction and genetic factors, resulting in a wide range of histological appearances and clinical features at presentation, including painless lymphadenopathy, B symptoms (weight loss > 10%, night sweats, body temperature > 38 °C), and so on [1]. The prognoses of NHL patients remain poor, while the 5-year overall survival (OS) rates have improved [2, 3]. Therefore, we posit that there may be other biomarkers potentially influencing the prognosis of NHL.

Programmed cell death ligand 1 (PD-L1), a 40 kDa type 1 transmembrane protein, can activate B, T cells, macrophages, and dendritic cells [4, 5]. It was first found by Chen et al in 1999 [6]. It was reported that PD-L1 co-stimulated T-cell proliferation and interleukin-10 secretion, which was considered to be involved in the negative regulation of cell-mediated immune responses [6]. Under normal physiological conditions, immune checkpoints maintain self-tolerance and protect tissues from damage when the immune system is responding to pathogenic infections [7, 8]. However, PD-L1, bound to programmed cell death 1 (PD-1), inhibits effector T cell function and activates immunosuppressive regulative T-cell function, resulting in tumors escaping under pathological conditions [9–11]. which is a major mechanism of tumor recurrence and drug resistance [12]. Moreover, clinical research inferred that patients who had overexpression of PD-L1 in tumors had improved clinical outcomes after taking checkpoint blockades [13].

Cumulative studies showed that PD-L1 or PD-1 could be used to determine prognosis in various cancers, such as melanoma, non-small cell lung cancer, kidney cancer [5], and classic Hodgkin lymphoma [14]. Some studies have also assessed the prognostic value of PD-L1 overexpression in NHL. However, the results were quite different. Thus, we aim to identify the problem through performing a meta-analysis.

## Methods

Our meta-analysis was conducted based on the Preferred Reporting Items for Systematic Reviews and Meta-Analyses (PRISMA) statement [15].

### Literature search

Four databases—PubMed, Cochrane Library, Web of Science, and Embase—were used to retrieve articles that investigated the prognostic value of PD-L1 overexpression in NHL. Additionally, we used the following terms for searches: “PD-L1,” “B7-H1,” “CD274,” “programmed cell death ligand 1,” “lymphoma,” “non-Hodgkin lymphoma,” “NHL,” “prognosis,” and “survival.” Articles published before January 2019 were included in the meta-analysis. We also performed a reference search.

### Selection of studies

Two independent reviewers evaluated all potential articles. All candidate articles had to meet the following criteria: (1) patients’ NHL diagnoses were histologically confirmed; (2) PD-L1 expression in lymphoid tissue was detected using immunohistochemistry (IHC); (3) hazard ratios (HRs) and 95% confidence intervals (CIs) could be directly obtained from the studies or calculated using data from the articles; and (4) the studies were full-text and written in English. Moreover, any disputes were solved via discussion.

### Data extraction and quality assessment

Two investigators independently extracted the data from articles. We extracted the following data: first author’s name, study country, publication year, subtype, sample size, cut-off value of PD-L1, HRs and 95% CIs for OS, PD-L1 positive number, follow-up period, treatment, Ann Arbor Stage and IHC antibodies. Furthermore, we contacted the author for original data if we were unable to calculate the effect size through the methods provided by Tierney [16]. We assessed these studies using the Newcastle–Ottawa Scale (NOS) [17], in which the score ranges from 0 to 9 points. We considered studies that received 6 points or above eligible for our meta-analysis. Any issues were resolved via discussion.

### Statistical analysis

HRs with 95% CIs were used to evaluate the correlation between PD-L1 overexpression and prognosis of NHL, and odds ratios (ORs) with 95% CIs were used to assess the association of PD-L1 overexpression with clinicopathological factors. Heterogeneity tests were performed using the I-squared statistics, and an I^2^ > 50% was considered significant. If heterogeneity was significant, we chose a random effect model to compute the pooled HR; otherwise, we selected a fixed effect model. Additionally, sensitivity analysis was used to assess the robustness of the pooled results, and publication bias was evaluated using Begg’s test. Subgroup analyses and meta-regression were performed due to significant heterogeneity. All the analyses were performed by STATA 12.0 software (STATA, College, TX) and Revman 5.3 (Revman the Cochrane, Collaboration, Oxford, England).

## Results

### Literature screening and characteristics

**Fig. 1:**
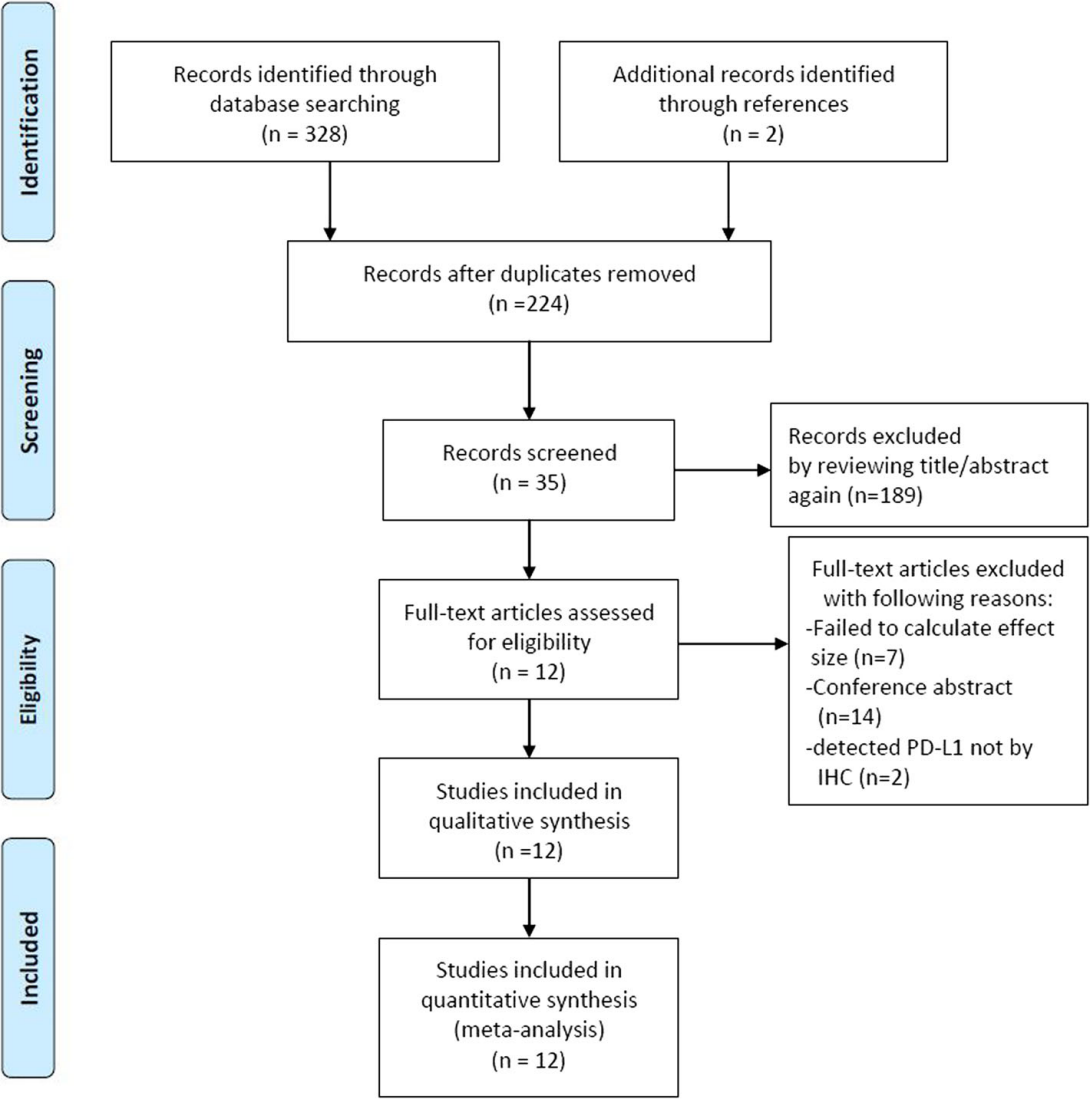
Flow chart of the included articles

The literature screening process is illustrated in Fig. 1. A total of 328 articles from the four databases and two articles from a manual reference search were initially selected. After removing duplicates, 224 studies remained. We excluded 189 articles after reviewing article abstracts. Next, seven articles were removed for failing to calculate the effect size; 14 studies were eliminated due to their being conference abstracts; and two studies were excluded because PD-L1 was not detected through IHC. Finally, altogether 12 articles encompassing 2321 patients were selected for the meta-analysis.

All characteristics of the studies are displayed in Table 1. Four studies were performed in China [18–21], four in Korea [22–25], two in Japan [26, 27], and one each was in the US [28] and Norway [29], respectively. The cut-off value was determined using the form of percentage except Cho’s, which ranged from 2 to 50%. According to the cut-off values, every article described the number of patients with PD-L1 overexpression. All studies referred to each disease stage according to Ann Arbor Staging except Bi’s. In addition, all studies were retrospective and reported the association between PD-L1 and OS. Patients in the studies had a histologically confirmed NHL diagnosis and subtype.

**Table 1:** Characteristics of studies

Study	Year	Sample size	Country	Tumor type	Median follow-up (range) (month)	Therapy	Stage	NOS	Cut-off	PD-L1+Number	Antibody			
											Company	Source	Type	Clone
Kiyasu	2015	1253	Japan	DLBCL	NA	C + T + R	I-IV	7	30%	461	abcam, UK	mouse	MAB	ab52587
Xing	2016	86	USA	DLBCL	21 (0.07–175)	C	I-IV	6	30%	14	Cell Signaling,USA	rabbit	MAB	E1L3N
Dong	2016	100	China	DLBCL	52.4 (1.5–89.1)	C	I-IV	7	5%	54	abcam, UK	rabbit	PAB	ab153991
Bi	2016	77	China	NK/T	38.0 (9.4–79.0)	C	I-II	8	38%	26	abcam, UK	rabbit	PAB	NA
Kim	2016	73	Korea	NK/T	20.6 (0.2–83.2)	C + S	I-IV	7	10%	41	Cell Signaling,USA	rabbit	MAB	E1L3N
Fang	2017	74	China	DLBCL	2.4–86.4	C + S	I-IV	8	10%	20	ZSGB-BIO	rabbit	MAB	SP142
Kwon	2015	126	Korea	DLBCL	52 (16–165)	C	I-IV	8	10%	77	Cell Signaling,USA	rabbit	MAB	E1L3N
Blaker	2016	38	Norway	FL	120 (15.6–408)	C + T	NA	6	2%	15	Spring Bioscience, Pleasanton,CA,USA	rabbit	MAB	SP142
Jo	2016	79	Korea	NK/T	52.4	C + R	I-IV	7	5%	63	R&D Systems,USA	mouse	MAB	NA
Hu	2017	204	China	DLBCL	52 (1–114)	C	I-IV	8	5%	100	Cell Signaling, USA	rabbit	MAB	NA
Cho	2017	76	Korea	PCNSL	20.2 (2.2–128.5)	C + T	NA	6	≥100 cells/HPF	10	Abcam, UK	rabbit	PAB	ab58810
Miyoshi	2016	135	Japan	ATLL	10.9 (0.03–114.8)	C + T + R	I-IV	8	50%	10	Abcam, UK	rabbit	MAB	ab174838

*DLBCL* diffuse large B cell lymphoma, *NK/T* NK/T cell lymphoma, *FL* follicular lymphoma, *PCNSL* primary central nervous system lymphoma, *ATLL* adult T cell lymphoma/leukemia, *C* Chemotherapy, *T* Transplantation, *R* Radiotherapy, *S* Surgery, *NOS* Newcastle–Ottawa Scale, *MAB* monoclonal antibody, *PAB* polyclonal antibody, *NA* not applicable, *NA* not applicable

### Association between PD-L1 overexpression and OS in NHL

**Fig. 2:**
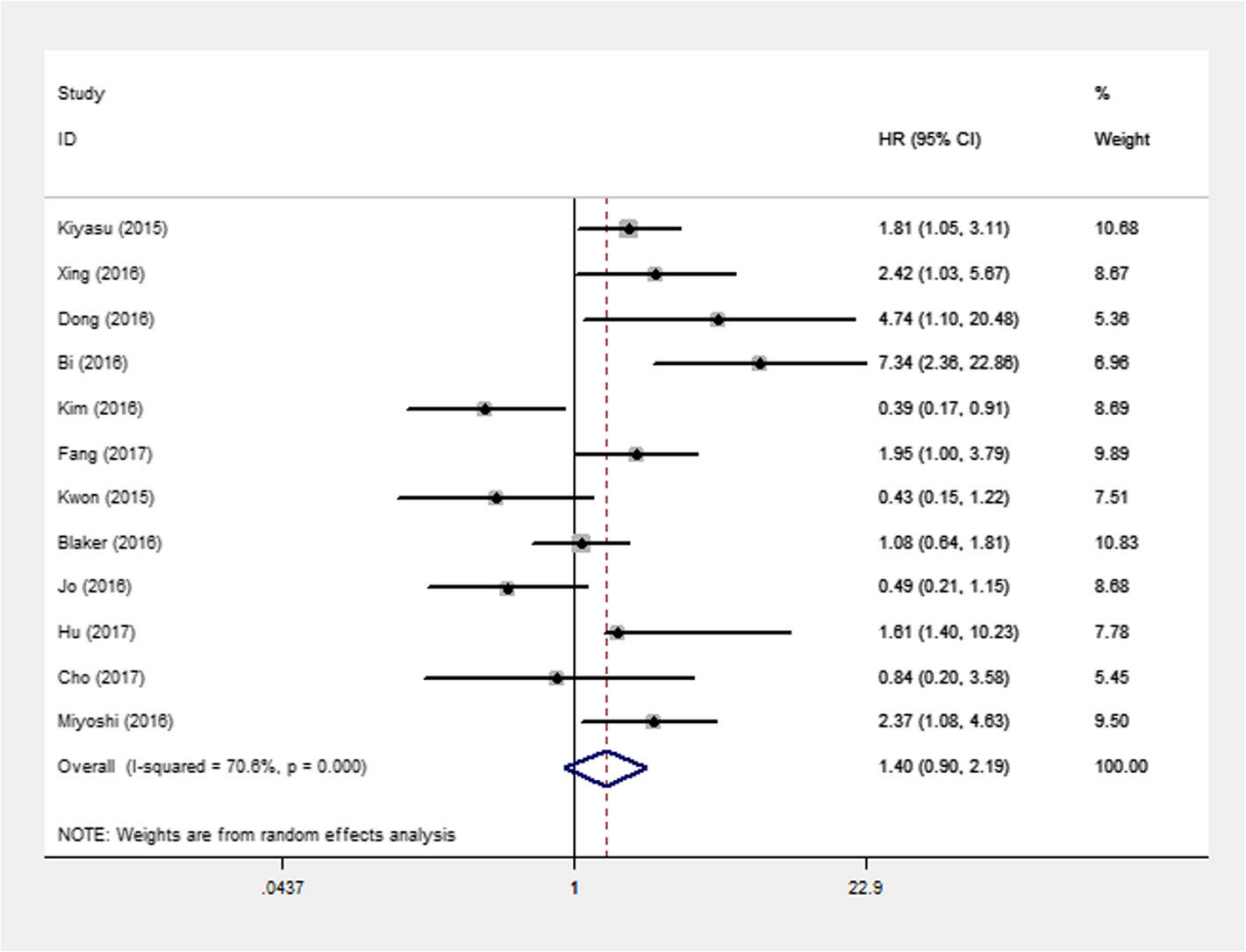
Forest plots of studies evaluating hazard ratio (HR) with 95% CI of PD-L1 for overall survival (OS) in NHL

We calculated a pooled HR of 1.40 (95% CI: 0.90–2.19; *P* = 0.137) for OS. The result indicated that PD-L1 overexpression was not associated with NHL prognosis. Significant heterogeneity, however, existed among the selected studies (I^2^ = 70.6%, *P* < 0.001; Fig. 2).

### Association of PD-L1 overexpression with OS in DLBCL

**Fig. 3:**
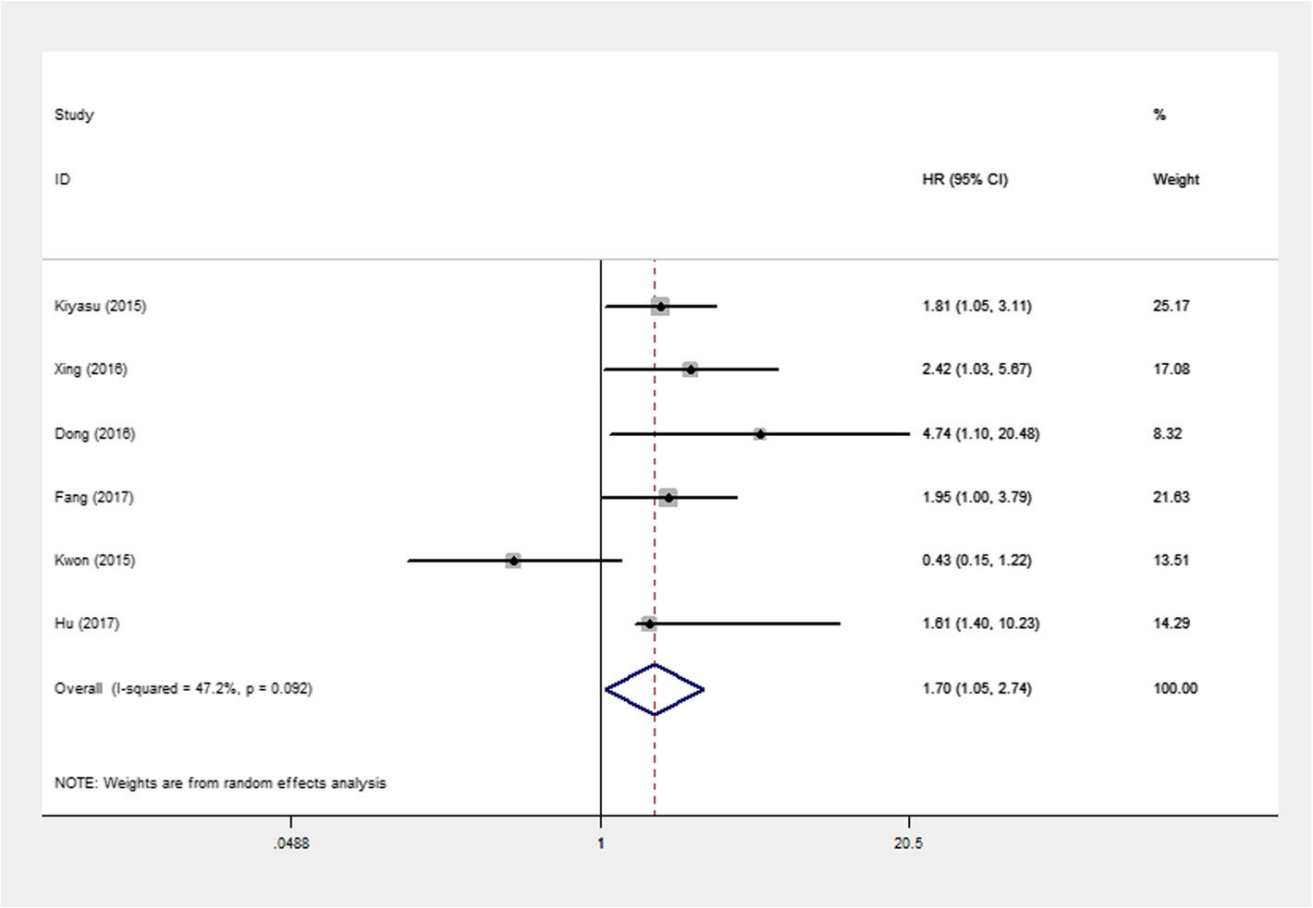
Forest plots of studies evaluating hazard ratio (HR) with 95% CI of PD-L1 for overall survival (OS) in DLBCL

DLBCL, accounting for 30–40% of NHL, is the most common subtype of NHL. There were 863 DLBCL patients from six articles in our study. A meta-analysis was performed that was designed to assess prognosis among DLBCL patients. The result showed that the pooled HR was 1.70 (95% CI: 1.05–2.74; *P* = 0.031) with I^2^ = 47.2% (Fig. 3). This indicated that PD-L1 overexpression potentially predicted a poor prognosis in DLBCL patients.

### Association between PD-L1 overexpression and clinicopathological characteristics

**Fig. 4:**
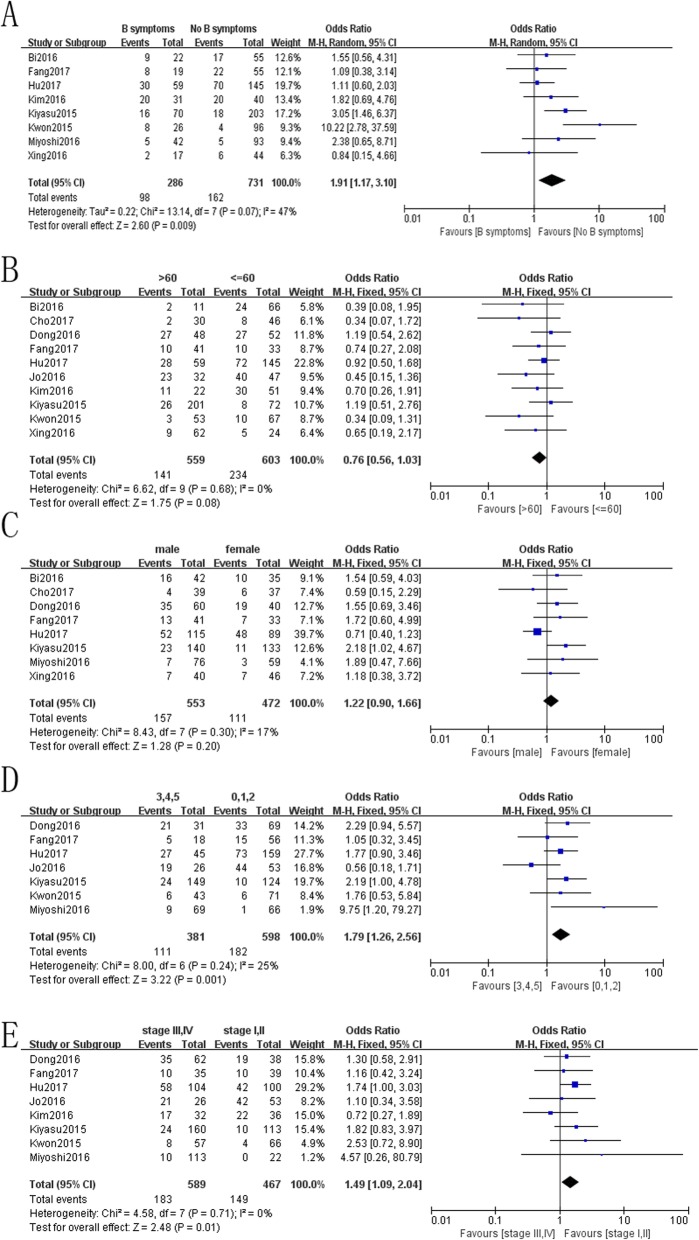
Forest plots for the association of PD-L1 overexpression with clinicopathological factors. **a** B symptoms; **b** age; **c** gender; **d** IPI score; **e** Ann Arbor stage

We also investigated the association of PD-L1 overexpression with clinicopathological characteristics. The results suggested that PD-L1 overexpression was more frequent in patients with B symptoms (OR = 1.91, 95% CI: 1.17–3.10; *P* = 0.09), stage III and IV (OR = 1.49, 95% CI: 1.09–2.04; *P* = 0.01) and international prognostic index (IPI) score of 3 to 5 points (OR = 1.79, 95% CI: 1.26–2.56; *P* = 0.001). However, there was no significant difference in the subgroups of gender and age (Fig. 4).

### Subgroup and sensitivity analysis

**Fig. 5:**
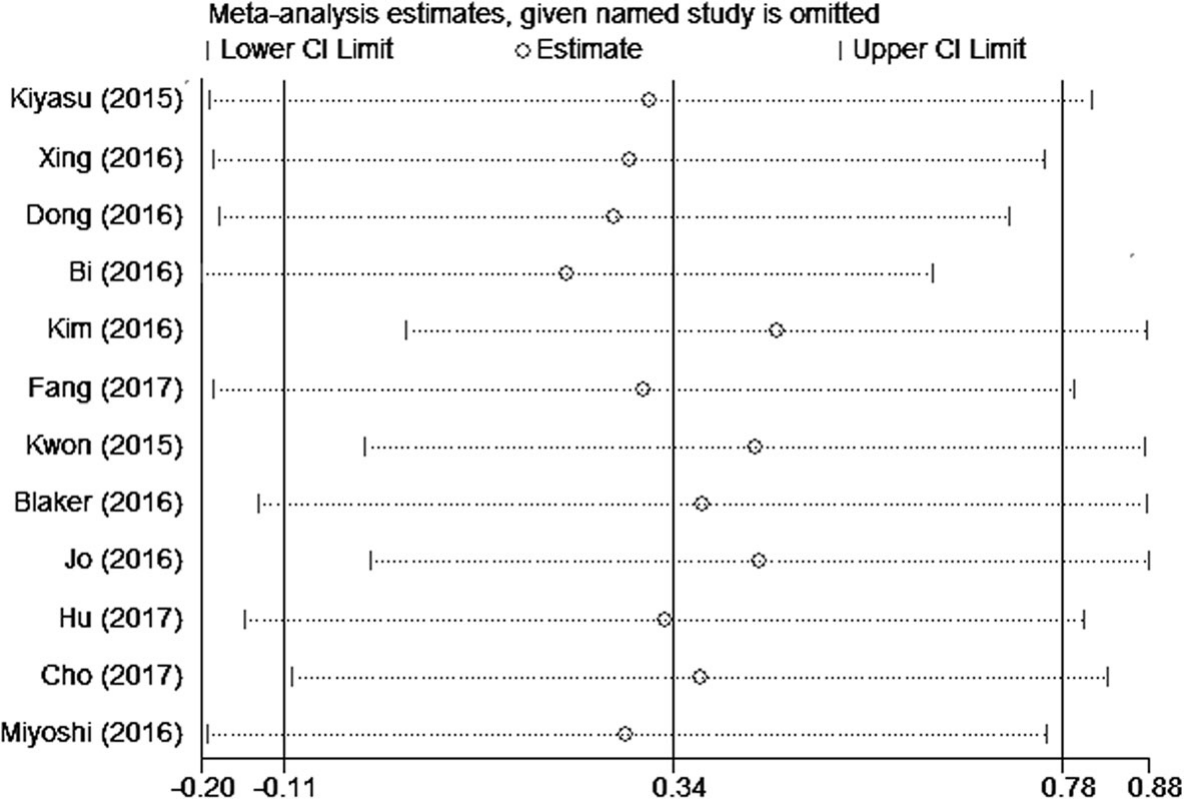
Sensitivity analysis on the correlation between PD-L1 and OS in NHL

**Fig. 6:**
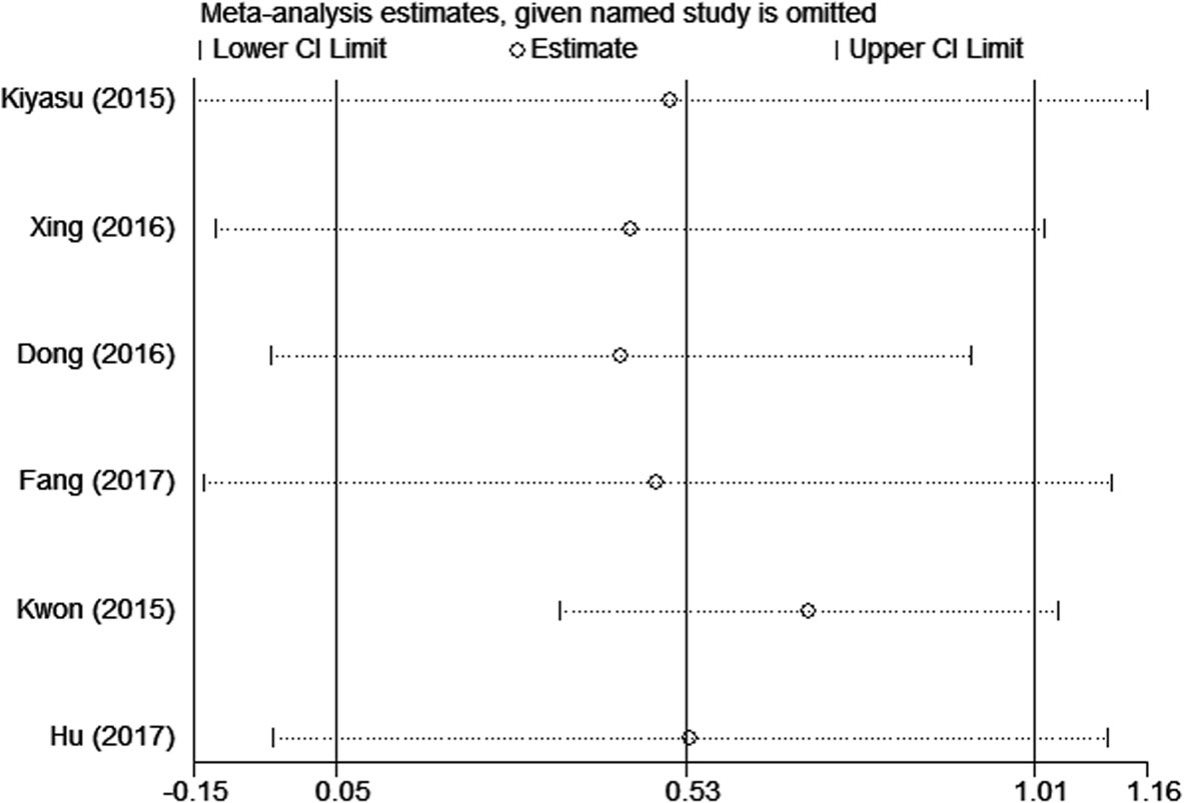
Sensitivity analysis on the correlation between PD-L1 and OS in DLBCL

Subgroup analyses were conducted by tumor type, country, sample size, cut-off value, therapy, antibody source, and type. Subgroup analysis by country showed HR of 2.86 (95% CI: 1.44–5.66; *P* = 0.003) in China, 1.99 (95% CI: 1.29–3.08; *P* = 0.002) in Japan, and 0.47 (95% CI: 0.29–0.77; *P* = 0.002) in Korea. In addition, when cut-off value ≥30%, HR was 2.54 (95% CI: 1.56–4.12; *P* < 0.001) with I^2^ = 37% (Table 2). Sensitivity analyses demonstrated that our pooled results were robust even when omitting anyone of the included studies by turn in NHL and DLBCL (Figs. 5 and 6).

**Table 2:** Subgroup analysis for OS

Subgroup	Number of studies	Number of patients	HR(95% CI)	*P* value	Heterogeneity
Location					
China	4	455	2.86 (1.44–5.66)	0.003	I^2^ = 45.1%; *P* = 0.141
Korea	4	354	0.47 (0.29–0.77)	0.002	I^2^ = 0%; *P* = 0.836
USA	1	86	2.42 (1.03–5.67)	0.042	/
Norway	1	38	1.08 (0.64–1.81)	0.771	/
Japan	2	1388	1.99 (1.29–3.08)	0.002	I^2^ = 0%; *P* = 0.557
Cut-off value					
≥ 30%	4	1627	2.54 (1.56–4.12)	< 0.001	I^2^ = 37%; *P* = 0.19
≤ 10%	7	694	0.98 (0.55–1.73)	0.938	I^2^ = 68.7%; *P* = 0.004
Tumor type					
DLBCL	6	1842	1.70 (1.05–2.74)	0.031	I^2^ = 47.2%; *P* = 0.092
NK/T	3	229	1.07 (0.21–5.59)	0.935	I^2^ = 89.3%; *P* < 0.001
FL	1	38	1.08 (0.64–1.81)	0.771	/
PCNSL	1	76	0.84 (0.20–3.55)	0.813	/
ATLL	1	136	2.37 (1.15–4.90)	0.020	/
Therapy					
Chemotherapy	5	1573	2.16 (0.85–5.49)	0.105	I^2^ = 73.6%; *P* = 0.004
Chemotherapy+other treatments	7	748	1.12 (0.69–1.84)	0.646	I^2^ = 68.5%; *P* = 0.004
Sample size					
≥ 100	5	1818	1.64 (0.90–3.01)	0.529	I^2^ = 57.8%; *P* = 0.05
< 100	7	503	1.26 (0.66–2.43)	0.480	I^2^ = 76.8%; *P* < 0.001
Antibody type					
MAB	9	2068	1.17 (0.75–1.83)	0.476	I^2^ = 68.4%; *P* = 0.001
PAB	3	253	3.23 (0.89–11.74)	0.075	I^2^ = 64%; *P* = 0.062
Antibody source					
Rabbit	10	989	1.52 (0.91–2.55)	0.212	I^2^ = 70.6%; *P* < 0.001
Mouse	2	1332	0.98 (0.27–3.52)	0.978	I^2^ = 84.5%; *P* = 0.011

*HR* hazard ratio, *CI* confidence interval, *DLBCL* diffuse large B cell lymphoma, *NK/T* NK/T cell lymphoma, *FL* follicular lymphoma, *PCNSL* primary central nervous system lymphoma, *ATLL* adult T cell lymphoma/leukemia, *MAB* monoclonal antibody, *PAB* polyclonal antibody

### Meta-regression analysis

Furthermore, meta-regression was performed for the source of heterogeneity in NHL. The results showed that sample size (*P* = 0.638), treatment (*P* = 0.229), location (*P* = 0.107), tumor type (*P* = 0.916), and cut-off value (*P* = 0.058) did not contribute to the heterogeneity.

### Publication bias

Begg’s test was used to assess the publication bias, which revealed no publication bias for either NHL (*P* = 0.880) nor DLBCL (*P* = 0.920).

## Discussion

This is a meta-analysis designed to investigate the relationship between PD-L1 overexpression and the prognosis of NHL. The association of PD-L1 overexpression with some clinicopathological factors was also evaluated. The pooled HR of 1.40 (95% CI: 0.90–2.19; *P* = 0.137) was calculated for 2321 patients from 12 studies, potentially indicating no significant correlation between PD-L1 and NHL prognosis. Nevertheless, the result suggested that PD-L1 overexpression was associated with poor prognosis in DLBCL patients. Figure 4 illustrates that patients with B symptoms, IPI scores of 3 to 5 points, and Ann Arbor Stage III or IV possessed overexpression of PD-L1.

Subgroup analysis and meta-regression showed no contribution to the heterogeneity in NHL. However, perhaps some problems contributed to the heterogeneity. Although IHC was used to detect PD-L1 protein in tumor cells in all studies, different studies adopted different procedures [30], antibody clones and thresholds [31]. Vranic et al. [32] suggested that anti-PD-L1 clones SP142 and SP263 exhibit an excellent concordance. Additionally, other confounding factors influence the expression of PD-L1. Studies [33, 34] indicated that anaplastic lymphoma kinase (ALK) up-regulates PD-L1 expression. Research also suggested that STAT3 regulates PD-L1 expression, and it was demonstrated that the inhibitor of STAT3 abrogated the expression of PD-L1 [35, 36]. It was also shown that tumor cells that overexpress PD-L1 protein have been frequently detected in EBV-positive lymphomas [20, 26, 37, 38].

The response to treatment is also not associated with the level of PD-L1 expression. Currently, PD-1 blockades are mostly employed clinically. Some clinical trials [39, 40] showed that patients with B-cell NHL indeed responded well to PD-1 blockades combined with rituximab. Zinzani et al. [41] found that PD-1 blockades used alone also benefited B-cell NHL patients. Two studies [42, 43] showed that PD-1 blockades helped relapsed or refractory NHL patients increase complete response rate. However, the level of PD-L1 expression in patients was quite different, and PD-L1was not even detected in some patients. These findings indicate that the level of PD-L1 expression is not associated with the prognosis of NHL patients.

Nevertheless, recent studies have uncovered the concrete functional mechanism of PD-L1 in DLBCL. PD-L1, bound to PD-1, caused phosphorylation of AKT, which urge m-TOR to activate its downstream molecules, such as P43-BP1 and P-P70S6K, finally resulting in proliferation and progression of malignant cells [19, 44, 45]. Theoretically, this explains why overexpression of PD-L1 causes short OS in DLBCL patients. Unfortunately, in other NHL subtypes, there is currently no such theory.

To the best of our knowledge, Zhao et al. [46] performed the first meta-analysis, which included 9 studies, to explore the relationship between PD-L1 overexpression and prognosis in NHL patients and concluded that PD-L1 overexpression has an association with poor prognosis in NHL and DLBCL but not with natural killer/T-cell (NK/T) lymphoma. We brought 12 studies with a total of 2321 patients into our meta-analysis and obtained conclusions that are different from Zhao et al.’s. In DLBCL and NK/T lymphoma (data not show), we reached the same conclusion as did Zhao et al. Yet, our conclusion regarding the overall result of NHL differs from that of Zhao et al*’*s due to our having included three more studies than they did. We also adopted two tools to conduct meta-analysis and did sub-analysis.

Several limitations, however, must be considered in interpreting our findings. First, the total sample size of the included studies was small. Second, other clinicopathological factors—such as EBV infection, tumor size, and central neutral system invasion—were not included in the analysis due to insufficient materials. Third, although we performed subgroup analysis by cut-off value, we did not know the best cut-off value for stratification of NHL patients in clinical management.

## Conclusions

In conclusion, our pooled results showed that overexpression of PD-L1 was not associated with OS in NHL patients; rather, it was associated with the subtype of DLBCL, indicating that PD-L1 could perhaps predict the prognosis of DLBCL. Furthermore, PD-L1 overexpression was associated with the clinicopathological factors of B symptoms, IPI score, and Ann Arbor Stage. Nevertheless, studies on other specific NHL subtypes using standardized immunological tests are needed to further explore the relationship between PD-L1 overexpression and prognosis of NHL.

## Data Availability

Not applicable.

## Abbreviations

CI: Confidence interval
DLBCL: Diffuse large B cell lymphoma
HR: Hazard ratio
IHC: Immunohistochemistry
IPI: International prognostic index
NHL: Non-Hodgkin lymphoma
NOS: Newcastle–Ottawa Scale
OR: Odds ratio
PD-1: Programmed cell death 1
PD-L1: Programmed cell death ligand 1
PRISMA: Preferred Reporting Items for Systematic Reviews and Meta-Analyses
